# HPR1 Is Required for High Light Intensity Induced Photorespiration in *Arabidopsis thaliana*

**DOI:** 10.3390/ijms23084444

**Published:** 2022-04-18

**Authors:** Zi Wang, Yetao Wang, Yukun Wang, Haotian Li, Zhiting Wen, Xin Hou

**Affiliations:** State Key Laboratory of Hybrid Rice, Hubei Hongshan Laboratory, College of Life Sciences, Wuhan University, Wuhan 430072, China; ziwang@whu.edu.cn (Z.W.); yetaowang@whu.edu.cn (Y.W.); alphahelix@whu.edu.cn (Y.W.); haotianli@whu.edu.cn (H.L.); zhitingwen@whu.edu.cn (Z.W.)

**Keywords:** photorespiration, photosynthesis, ROS, high light intensity

## Abstract

High light intensity as one of the stresses could lead to generation of large amounts of reactive oxygen species (ROS) in plants, resulting in severe plant growth retardation. The photorespiration metabolism plays an important role in producing and removing a variety of ROS, maintaining the dynamic balance of the redox reaction, and preventing photoinhibition. *Arabidopsis* hydroxypyruvate reductase 1 (HPR1) is a primary metabolic enzyme in the photorespiration cycle. However, the role of HPR1 in plants response to high light is not clear. Here, we found that the expression of *HPR1* could be induced by high light intensity. The growth and photosynthetic capacity of *hpr1* mutants are seriously affected under high light intensity. The absence of HPR1 suppresses the rates of photorepair of Photosystem II (PSII), aggravates the production of ROS, and accelerates photorespiration rates. Moreover, the activity of ROS scavenging enzymes in the *hpr1* mutants is significantly higher. These results indicate that HPR1 is involved in plant response to high light intensity and is essential for maintaining the dynamic balance of ROS and photorespiration.

## 1. Introduction

In the natural environment, plants are exposed to comprehensive and complicated conditions. Some environmental stresses may cause excessive generation of reactive oxygen species (ROS) in plant cells, which are major causes of low crop yield worldwide [1,2,3,4,5]. The term ROS encompasses oxygen free radicals, such as superoxide anion radicals (O_2_^·−^), hydroxyl radicals (·OH), and nonradical oxidants such as hydrogen peroxide (H_2_O_2_) and singlet oxygen (^1^O_2_) [6]. High light intensity and high temperature would cause the generation of ^1^O_2_; air pollutants like O_3_, SO_2_ and lead to H_2_O_2_ production. Specifically, ROS could generate oxidative damage to membranes (lipid peroxidation) and proteins, and could even result in the oxidative destruction of the cells in a process termed oxidative stress [1]. Stress-induced ROS generation is counteracted by enzymatic antioxidant systems that include a variety of scavengers, such as catalase (CAT) and superoxide dismutase (SOD) [7]. In photosynthetic tissues (leaves), chloroplasts are the major sources of ROS generation in plants. High light can lead to an overexcitation of the photosynthetic apparatus [8] and decrease the photosynthetic efficiency.

Photorespiration is closely related to the generation and scavenging of ROS. Photorespiration is a carbon metabolism pathway evolved by photosynthetic organisms in response to elevated O_2_ concentrations in the air. RuBisCO catalyzes both carboxylation, which is photosynthesis, and oxygenation, which is photorespiration. Photorespiration always occurs along with photosynthesis, which means that oxygen-emitting photosynthetic organisms absorb oxygen, consume organisms and release CO_2_ under light. Although the function of photorespiration is still controversial, it seems to be widely accepted that photorespiration affects many metabolic processes, such as energy metabolism, photosystem II function, carbon metabolism, nitrogen assimilation and respiration [9]. Photorespiration is a complex metabolic process that requires the synergistic action of chloroplasts, mitochondria and peroxisomes. It has been reported that organelles such as chloroplasts, mitochondria and peroxisomes with highly oxidized metabolic activity or with an intense rate of electron flow are a major source of ROS in plant cells [7]. Peroxisomes are small and single membrane-delimited organelles that house numerous oxidative reactions connected with metabolism and development. Peroxisomes are probably the major sites of intracellular ROS production [10,11]. Therefore, peroxisomes are one of the major sites of photorespiration, as well as one of the major sites of ROS generation.

The functions of many photorespiratory core members as well as their inner links have been revealed [12]. The conversion of hydroxypyruvate (HP) into glycerate (GLY) was a specific reaction that was required in photorespiratory [13]. Hydroxypyruvate reductase 1 (HPR1) catalyzes the conversion of HP to GLY in peroxisomes. Most photorespiratory mutants were lethal in normal air, whereas *hpr1* mutants could survive. Therefore, we noticed that three enzymes localized in different organelles catalyzed the same reaction of HP to GLY. The other two isoenzymes, hydroxypyruvate reductase 2, (HPR2) was located in the cytoplasm, and the location of hydroxypyruvate reductase 3 (HPR3) was unknown [14]. What’s more, the relationships among them were largely unrevealed.

Here, we isolated *hydroxypyruvate reductase 1* (*hpr1*) mutants. Under high light intensity stress, the loss of HPR1 disrupted photosynthetic efficiency and caused serious photoinhibition. Also, there were excessive ROS and active scavenging enzymatic systems in the *hpr1* mutants under high light intensity, as well as high contents of photorespiratory intermediates, suggesting that HPR1 might work as a key photorespiratory protein in regulating oxidative damage under extreme environments.

## 2. Results

### 2.1. The Expression of HPRs Is Induced by High Light

**Figure 1:**
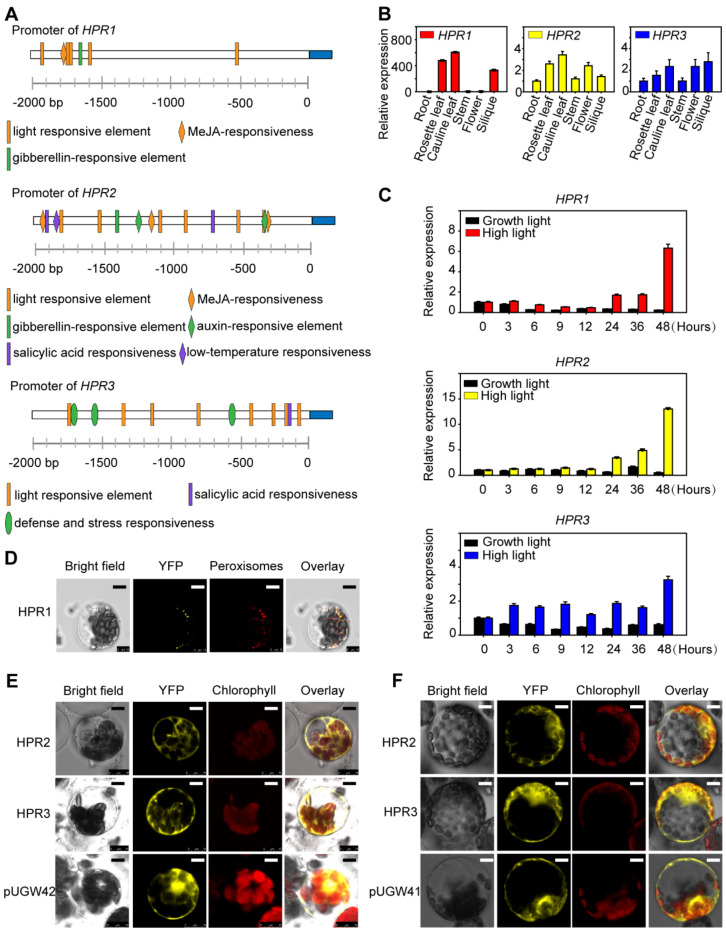
The expression pattern analysis of *HPR1*, *HPR2* and *HPR3*. (**A**) *Cis*-acting regulatory elements analysis. The 2-kb DNA fragments at upstream of the ATG starting code of the *HPR1*, *HPR2* and *HPR3* genes were analyzed using PlantCARE. (PlantCARE address: http://bioinformatics.psb.ugent.be/webtools/plantcare/html/, accessed on 17 December 2021). (**B**) Expression patterns of *HPR1*, *HPR2* and *HPR3* in various organs of four-week-old *Arabidopsis* analyzed via quantitative RT-PCR. Data shown are means ± SD, n = 3, with three independent replicates. *ACTIN* was used as an internal control. (**C**) Expression of *HPR1*, *HPR2* and *HPR3* in leaves of *Arabidopsis* plants exposed to continuous high light intensity 350 µmol·m^−2^·s^−1^. Values represent means ± SD, n = 3, with three independent replicates. *ACTIN* was used as an internal control. (**D**) Subcellular localizations of HPR1. Confocal microscopic images of YFP-HPR1 fusion proteins expressed transiently in protoplasts of *Arabidopsis* wild types. Proteins fused with mCherry, which located in the peroxisome as the marker. Bars = 10 µm. (**E**) Subcellular localizations of HPR2 and HPR3. Confocal microscopic images of YFP-HPRs fusion proteins expressed transiently in protoplasts of *Arabidopsis* wild types. Free YFP of pUGW42 was used as a control. YFP fluorescence, chloroplast autofluorescence and merged images are shown. Bars = 10 µm. (**F**) Subcellular localizations of HPR2 and HPR3. Confocal microscopic images of HPRs-YFP fusion proteins expressed transiently in protoplasts of *Arabidopsis* wild types. Free YFP of pUGW41 was used as a control. YFP fluorescence, chloroplast autofluorescence and merged images are shown. Bars = 10 µm.

HPR1 is an important metabolic enzyme in the photorespiration process which has another two isoenzymes, HPR2 and HPR3 [13]. To understand the regulation of gene expression, we performed a promoter analysis to identify regulatory *cis*-elements using PlantCARE. As shown in Figure 1A, manifold *cis*-regulatory elements were identified in 2 kb DNA fragments upstream of the ATG start code. The results exhibited that there was one *cis*-element (CGTCA motif) involved in the MeJA response, one *cis*-element (P-box: CCTTTTG) related to the gibberellin response, and mostly *cis*-elements response to light in the *HPR1* promoter (Figure 1A). Similarly, several light-responsive *cis*-elements were in the *HPR2* and *HPR3* promoters (Figure 1A), far beyond other stress-responsive elements. To further investigate the function of these three enzymes, we examined the expression patterns of *HPRs* genes. Gene expression levels analyzed by qRT-PCR showed that *HPR2* and *HPR3* expression levels in different organs were close, and the expression was higher in leaf, flower and silique than in root and stem no more than threefold (Figure 1B). However, *HPR1* was expressed to different extents in all aboveground tissues, which had more predominant mRNA accumulation in leaf and silique than root, over 600 times (Figure 1B). We could see from charts that *HPR1*, *HPR2* and *HPR3* had predominant mRNA accumulation in photosynthetic tissues such as leaves and reproductive tissues such as siliques (Figure 1B), whereas there was minimal expression in roots for these three genes (Figure 1B). We inferred that these enzymes were regulated by light on account of its higher expression in photosynthetic tissues such as leaves and minimal expression in roots. This observation suggested that these three enzymes were probably regulated by light. Therefore, to explore whether they responded to high light environment, we examined the expression levels of these genes in WT plants after high light (350 µmol·m^−2^·s^−1^) treatment. When growing under normal light intensity, the expression of *HPR1*, *HPR2* and *HPR3* in WT all fluctuated within a normal range. Whereas qRT–PCR results showed that after high light exposition the expression of all the genes were up-regulated as the time was extended, finally increasing to 6, 13 and 3 times higher than that in the control group at the point time of the 48th hour, respectively (Figure 1C). Taken together, *HPR1*, *HPR2* and *HPR3* probably responded to high light.

We fused the yellow fluorescent protein (YFP) to determine subcellular localizations of HPR1, HPR2 and HPR3 experimentally. *HPR1* had C-terminal peroxisomal targeting signal-SKL [15]. Therefore, we fused YFP to the N-terminal of HPR1. YFP-HPR1 and the plastid of px-rk CD3-983, which marked peroxisomes, were expressed simultaneously in protoplasts of *Arabidopsis thaliana* WT and further investigated by confocal laser scanning microscope. The results showed that YFP- HPR1 co-localized with px-rk CD3-983 in peroxisomes (Figure 1D). To rigorously determine the locations of HPR2 and HPR3, we transformed CDS of HPR2 and HPR3 into pUGW41, in which YFP at the C-terminal. Also, we transformed CDS of HPR2 and HPR3 into pUGW42, in which YFP at the N-terminal. Both results suggested that HPR2 and HPR3 resided in the cytoplasm (Figure 1E,F).

### 2.2. HPR1 Functions in Plant Response to High Light Intensity

**Figure 2:**
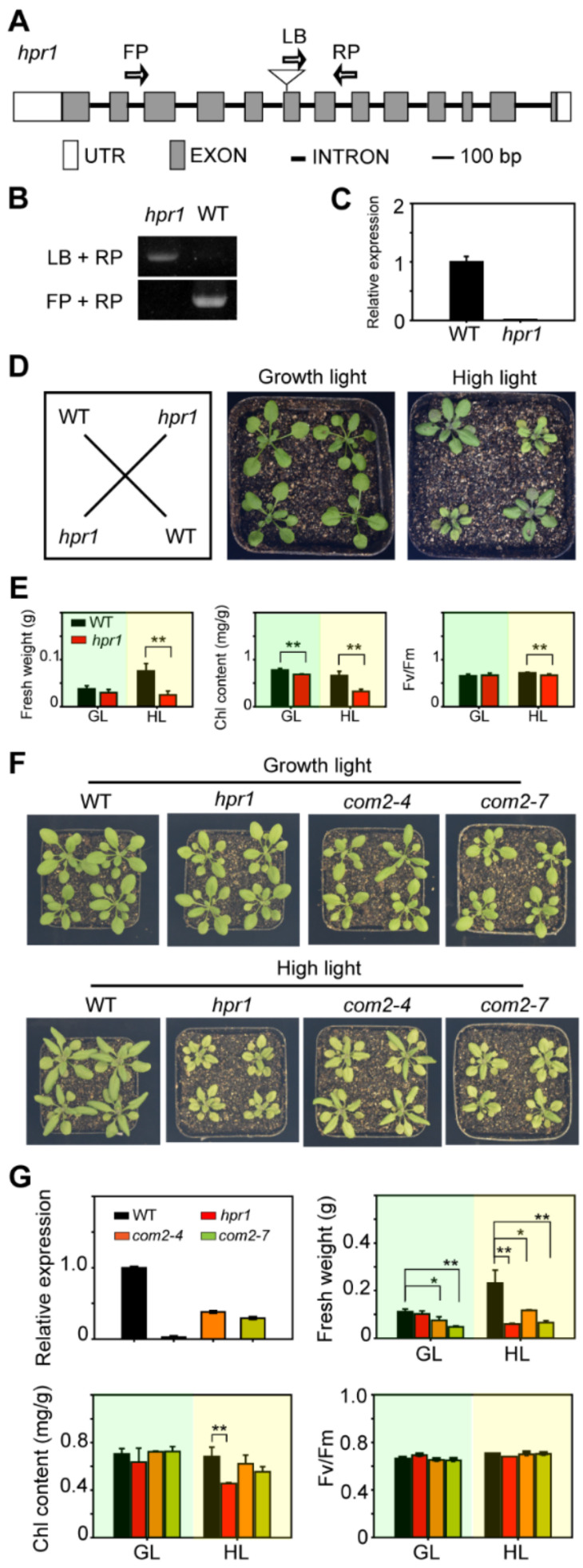
Identification of *hpr1*. (**A**) Genomic structure model of *HPR1*. Gray rectangles show the open reading frame, the black line shows intron, white rectangles show untranslated regions, and the triangle represents T-DNA insertion. (**B**) PCR analysis of genomic DNA from the WT and *hpr1* plants. LB, RP and FP indicate primers which locations are shown in (**A**). (**C**) The expression of *HPR1* in WT and *hpr1* plants was analyzed by qRT-PCR. (**D**) Phenotypes of four-week-old WT and *hpr1* mutants. We transferred three-week-old plants grown in soil to growth light (GL, 80 µmol·m^−2^·s^−1^) and high light (HL, 350 µmol·m^−2^·s^−1^) for another week, respectively. (**E**) Fresh weight (left), chlorophyll content (middle), and chlorophyll fluorescence (right) of WT and *hpr1* plants grown in soil. They were treated with the same light intensity in (**D**), respectively. Data shown are means ± SD, n = 3, with three independent replicates. Asterisks show significant differences compared to WT: ** *p* < 0.01 (Student’s *t* test). (**F**) Molecular complementation assay by expressing full length CDS of *HPR1* in the *hpr1* mutants. WT, *hpr1*, and two complementary lines treated by growth light with 80 µmol·m^−2^·s^−1^ and high light intensity with 350 µmol·m^−2^·s^−1^ for one week. (**G**) The *HPR1* gene expression level, fresh weight, chlorophyll content and chlorophyll fluorescence of WT, *hpr1*, and two complementary lines grown under growth light (GL, 80 µmol·m^−2^·s^−1^) and high light intensity (HL, 350 µmol·m^−2^·s^−1^). Data shown are means ± SD, n = 3, with three independent replicates. Asterisks show significant differences compared to WT: * *p* < 0.05; ** *p* < 0.01 (Student’s *t*-test).

The extremely elevated expression induced by high light intensity of *HPRs* led us to test whether these genes function in plants responding to high light intensity. We identified two T-DNA insertional mutants, *hpr1* and *hpr3*. For *At1g68010*, in *hpr1*, the T-DNA insertion localized in the sixth exon of *HPR1* (Figure 2A), and both DNA strands had T-DNA inserted (Figure 2B). The *HPR1* mRNA could not be detected (Figure 2C). In *hpr3*, T-DNA inserted in the second exon of *HPR3* (Supplementary Figure S1A), both strands of DNA had T-DNA inserted (Figure S1B), and the expression of *HPR3* was almost undetectable (Figure S1C). The mutants and WT were cultivated in a growth chamber at 22 °C, and treated under growth light intensity (80 µmol·m^−2^·s^−1^) and high light intensity (350 µmol·m^−2^·s^−1^) conditions (16-h light/ 8-h dark) for one week. After treatments, there was no visible difference between WT and the mutants under growth light conditions. However, under high light intensity, compared with WT, the *hpr1* mutants were smaller and pale yellow (Figure 2D), while *hpr3* grew as well as WT (Figure S1D). The results indicated that HPR1 played an important role in plant adaptation to high light. The obvious phenotype stimulated us to explore the crucial mechanisms of HPR1’s response to high light intensity.

To see more details, we measured the fresh weight, chlorophyll content, and photosynthetic efficiency of WT and *hpr1*. The results showed that under growth light intensity there was no difference in fresh weight and maximum light quantum efficiency between *hpr1* and WT, while the chlorophyll content of the mutant was significantly lower than that of the wild type (Figure 2E). Under high light intensity, the fresh weight of *hpr1* was significantly lower than WT, which was ^1^/_3_ of WT (Figure 2E, left), and the maximum light quantum efficiency of *hpr1* was also significantly lower than that of WT, which was 0.72 under high light intensity, while *hpr1* was only 0.66 (Figure 2E, right). The chlorophyll content of the mutant was also significantly lower than that of the wild type, and the difference was even more significant under high light intensity, only half of that of the wild type (Figure 2E, middle). These results suggested that the *hpr1* mutants had serious defects in growth and photosynthesis under high light intensity.

To confirm whether the phenotypic change in the *hpr1* mutant was caused by disruption of HPR1, we performed a complementation assay by expressing a 1164 bp wild-type *HPR1* coding region driven by a 35S promoter in the mutants. Two complementary lines with different expression levels of HPR1 were used to verify that growth retardation under high light was caused by HPR1 defects. *com2-4* achieved 38% and *com2-7* achieved 29% of the WT in *HPR1* expression levels (Figure 2G). Under high light intensity, the chlorophyll content of these two complementary lines were much higher than the *hpr1* mutants (Figure 2F,G). As shown in Figure 2F,G, the fresh weight of WT under high light was twofold greater than that under growth light, while the fresh weight of *hpr1* mutant under high light decreased 50% compared to that under growth light, suggesting that the *hpr1* mutant was sensitive to high light. The fresh weight of complementary lines under high light increased by 1.5 times and 1.3 times compared with that under growth light, suggesting that the retardant phenotypes and physiological function of *hpr1* under high light were complemented by HPR1 in the transgenic plants (Figure 2F).

### 2.3. Loss of HPR1 Affects Photosynthetic Efficiency

**Figure 3:**
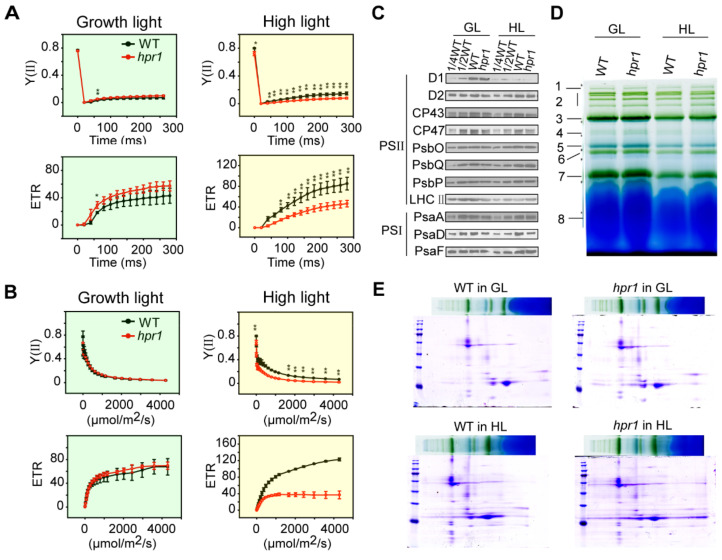
Photosystem analysis in WT and *hpr1* mutants. (**A**) Slow chlorophyll fluorescence induction kinetics of four-week WT and *hpr1* cultivated at growth light (80 µmol·m^−2^·s^−1^) and high light (350 µmol·m^−2^·s^−1^). Y(II), quantum yield of PSII photochemistry; ETR, electron transport rate through PSII. Values shown are means ± SD, n = 3, with three independent replicates. Asterisks show significant differences compared to WT: *, *p* < 0.05; ** *p* < 0.01 (Student’s *t*-test). (**B**) Light response curves of PSII of four-week-old WT and *hpr1* cultivated at growth light of 80 µmol·m^−2^·s^−1^ and high light intensity of 350 µmol·m^−2^·s^−1^. Values shown are means ± SD, n = 3, with three independent replicates. Asterisks show significant differences compared to WT: * *p* < 0.05; **, *p* < 0.01 (Student’s *t*-test). (**C**) Western blot analysis of photosystem proteins in four-week-old WT and *hpr1* mutant plants under growth light (GL, 80 µmol·m^−2^·s^−1^) and high light intensity (HL, 350 µmol·m^−2^·s^−1^). Immunoblot analysis was performed with antibodies against the indicated thylakoid membrane proteins. (**D**) Blue native gel analysis of photosynthetic complexes of four-week-old WT and *hpr1* mutant plants. Annotation of the different complexes: 1, NDH-PSI; 2, PSII supercomplexes; 3, PSI monomer, PSII dimer and PSII monomer with LHCII trimers; 4, PSI monomer and CF_1_ complex; 5, PSII monomer; 6, LHCII assembly; 7, LHCII trimers; 8, LHCII monomers. (**E**) Thylakoid proteins separated by BN gel in (**D**) were further subjected to the second dimension SDS PAGE.

Based on the etiolated phenotypes, the reduced chlorophyll contents and photosynthetic efficiency under high light intensity, we proposed that the lack of HPR1 affected the photosystem. To determine whether the absence of HPR1 protein affected the function of photosynthesis, various photosynthetic parameters were examined. Chlorophyll fluorescence kinetics could reflect the real-time process of biological electron transfer in vivo, which was closely related to the state of plant photosynthesis. We measured PSII photosynthetic parameters of plants cultured under a growth light intensity of 80 µmol·m^−2^·s^−1^ and a high light intensity of 350 µmol·m^−2^·s^−1^ for one week by pulse-amplitude-modulation with a chlorophyll fluorimeter PAM-2500. Observing the response process of the photosynthetic system of samples from dark to light, and the curve of this change was the chlorophyll fluorescence induction curve—OJIP curve [16]. Y(II) represents the actual photosynthetic efficiency of PSII, reflecting the current actual light energy conversion efficiency of PSII, and ETR represents the electron transfer rates of PSII. All the electrons accumulated at PQ were transferred away, and all the reaction centers of PSII were in an open state. Then the light was turned on and measured. As shown in Figure 3A, Y(II) and ETR of the mutant and wild type growing under growth light intensity showed no difference, the Y(II) values of WT and *hpr1* increased slowly and finally stabilized at 0.07 and 0.09, respectively (Figure 3A). ETR values also increased steadily, eventually stabilizing at 42 and 57, respectively (Figure 3A). Under high-light treatment, the Y(II) and ETR values of the *hpr1* mutants were significantly lower than WT with the extension of induction time, and the ETR values of *hpr1* mutants were always rising at a constant rate below WT curve (Figure 3A).

The light response curve shows the changes of photosynthetic rates of plants with the increase of light intensity, which reflects the potential photosynthetic activity. With the increase of light intensity, Y(II) and ETR of the plants grown under growth light intensity had no difference (Figure 3B). In the range of 0–500 µmol·m^−2^·s^−1^ light intensity, Y(II) decreased sharply, and then gradually decreased to 0 (Figure 3B). Similarly, ETR rose rapidly to 40 and then gradually to 70 (Figure 3B). Y(II) and ETR of the *hpr1* mutants treated with high light intensity were significantly lower than that of WT (Figure 3B). The ETR value of the mutant was always significantly lower than that of the wild type, and the difference exacerbated with the increase of PAR (Figure 3B). These data indicated that the loss of HPR1 caused severe damage to PSII under high light.

Further experiments were performed to detect photosynthetic protein accumulation. Immunoblotting against various chloroplast proteins, PSII proteins: D1, D2, CP43, CP47, PsbP, PsbQ, PsbO and LHCII; PSI proteins: PsaA, PsaD and PsaF. The results showed that there was no difference in the accumulation of photosynthetic proteins in the *hpr1* mutants grown under growth light intensity or high light intensity, compared with WT, indicating that the deficiency of HPR1 did not affect photosynthetic protein accumulation (Figure 3C). We then performed Blue-Native PAGE and Second Dimension SDS–PAGE experiments to investigate whether the lack of HPR1 protein affected the assembly of photosynthetic complexes. The native gels revealed no detectable differences in supercomplex formation between WT and *hpr1* mutants (Figure 3D). Further analysis of the second-dimension results showed that the structure of the complex was similar in WT and mutants (Figure 3E).

### 2.4. Loss of HPR1 Accelerates PSII Photoinhibition by Suppressing Photorepair

Light is a necessary energy source to drive the process of photosynthesis. When light intensity exceeds the energy required for photosynthesis, the photosynthetic mechanism will be destroyed, especially PSII, which will lead to photoinhibition. Therefore, photoinhibition is inevitable [17]. However, the degree of photoinhibition depends on the balance between PSII core photodamage and photorepair mechanisms [18]. The photorespiratory pathway has been shown to be one of the mechanisms responsible for protecting PSII from photoinhibition [19,20,21,22].

**Figure 4:**
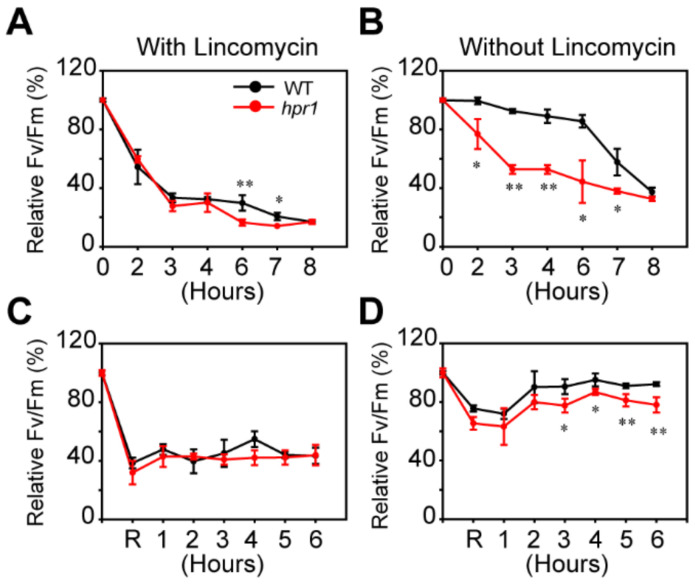
PSII photoinhibition analysis. (**A**,**B**) Photoinhibition of PSII in wild-type and *hpr1* mutants was examined. Detached leaves from wild types and mutants were exposed to light at 1000 µmol·m^−2^·s^−1^ in the absence or presence of 1 mM lincomycin. The maximal photochemical efficiency of PSII (*F_v_*/*F_m_*) was measured after dark adaptation for 15 min. Data shown are means ± SD, n = 3, with three independent replicates. Asterisks show significant differences compared to WT: * *p* < 0.05; ** *p* < 0.01 (Student’s *t*-test). (**C**,**D**) Recovery of *F_v_*/*F_m_* after photoinhibition in wild-types and *hpr1* mutants. Detached leaves from wild types and mutants were exposed to light at 1000 µmol·m^−2^·s^−1^ in the absence or presence of 1 mM lincomycin until the PSII activity was reduced to 60%, and then subsequently, shifted to low light (60 µmol·m^−2^·s^−1^) to recover. Data shown are means ± SD, n = 3, with three independent replicates. Asterisks show significant differences compared to WT: * *p* < 0.05; ** *p* < 0.01 (Student’s *t*-test).

To investigate whether the *hpr1* mutants could work in PSII photoinhibition, we illuminated detached leaves from four-week-old WT and mutant plants with heterochromatic light at the intensity of 1000 µmol·m^−2^·s^−1^ with or without lincomycin. Lincomycin could inhibit chloroplast protein synthesis. So, we monitored the process of photodamage by inhibiting PSII repair with lincomycin. The *F_v_*/*F_m_* values for WT and the *hpr1* mutants decreased to 16% and 15% after 6-h high light, respectively, with lincomycin treatment (Figure 4A). Without lincomycin treatment, the *F_v_*/*F_m_* dropped more quickly in *hpr1* mutants than in WT at each time point (Figure 4B), which indicated that *hpr1* was more sensitive to high light and HPR1 may be involved in recovery. Then, we performed a recovery experiment at low light intensity (60 µmol·m^−2^·s^−1^) for 6 h after PSII activity decreased to 60% under high light of 1000 µmol·m^−2^·s^−1^. As expected, without lincomycin, the recovery rates of the *hpr1* mutants were slower than those of WT (Figure 4D), suggesting that the loss of HPR1 accelerated photoinhibition by suppressing the photorepair of PSII.

### 2.5. HPR1 Functions in High Light Induced ROS Production

**Figure 5:**
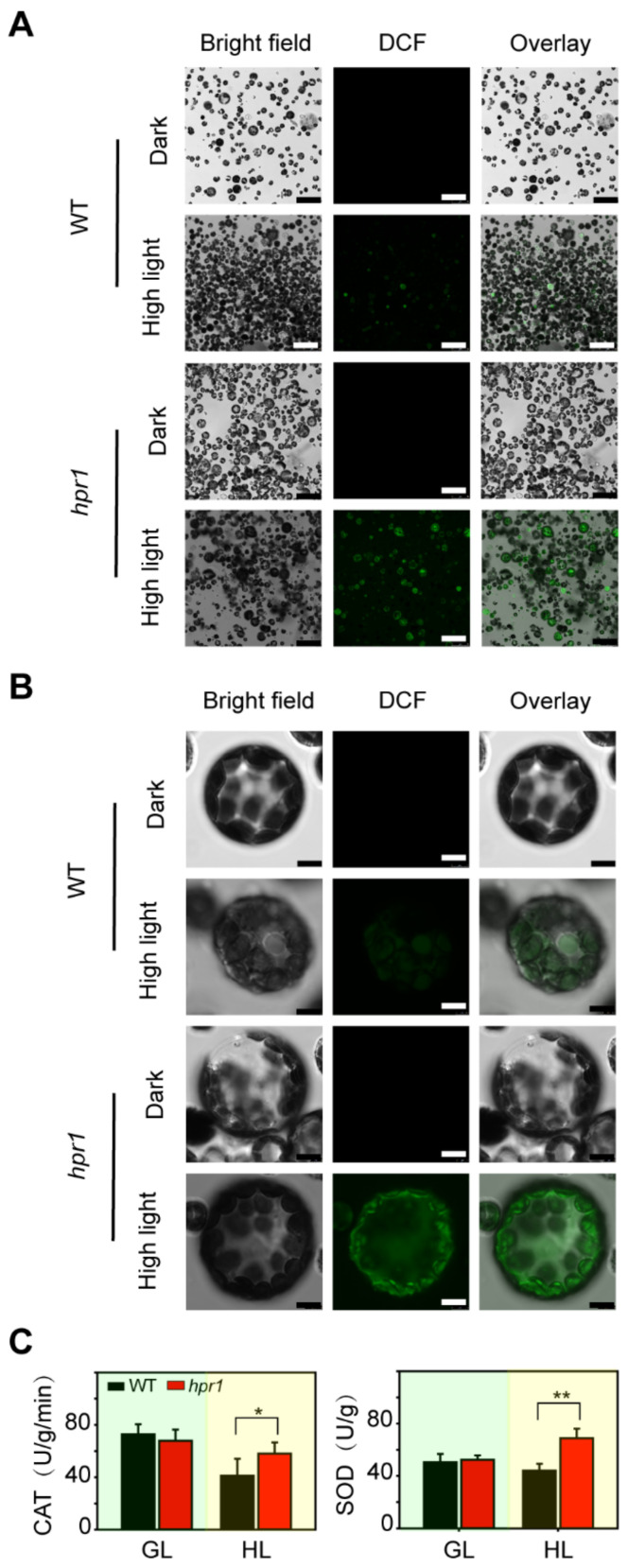
Detection of ROS production and removal. (**A**,**B**) Detection of ROS production. Protoplasts from WT and *hpr1* mutants were treated with or without high light (1000 µmol·m^−2^·s^−1^) for 30 min, incubated with H_2_DCFDA (at a final concentration of 5 µM), and observed using LCSM as described in the Materials and Methods. (**C**) Superoxide dismutase (SOD) and catalase (CAT) activities in WT and *hpr1* grown under growth light (GL, 80 µmol·m^−2^·s^−1^) and high light (HL, 350 µmol·m^−2^·s^−1^). Values shown are means ± SD, n = 3, with three independent replicates. Asterisks show significant differences compared to WT: * *p* < 0.05; ** *p* < 0.01 (Student’s *t* test).

High light is a factor of abiotic stress that can cause ROS. We observed that high light seriously destroyed PSII activity in the *hpr1* mutants, and photorepair experiments showed that *hpr1* mutants exhibited obvious photoinhibition. When light energy was excessive, photosynthetic pigments produced a large amount of ROS [2]. Therefore, we examined the generation and removal of ROS in the *hpr1* mutants. To analyze ROS distribution, the protoplasts of WT and mutants were incubated with H_2_DCFDA and illuminated for 30 min under 1000 µmol·m^−2^·s^−1^. We then detected the oxidation of H_2_DCFDA into DCF with fluorescence. Before high light illumination, ROS could not be detected in either WT or the mutant (Figure 5A,B). After high light treatment for 30 min, ROS generated in protoplasts derived from WT and mutants. *hpr1* protoplasts were oxidized and generated more ROS than WT protoplasts (Figure 5A). AS shown in Figure 5B, the enlarged images could provide more details. In individual protoplasts, the intensity and area of fluorescence in the mutants were significantly greater than those in the wild types (Figure 5B), indicating that the loss of HPR1 led to more high light induced ROS generation in the mutants.

In addition to the generation of ROS in plants, we also compared the ROS scavenging ability of WT and mutant under high light conditions. CAT, which reduces H_2_O_2_ into H_2_O, and SOD, which removes oxygen radicals, were the major ROS scavenging enzymes. The SOD and CAT were extracted from WT and *hpr1* grown under growth light (80 µmol·m^−2^·s^−1^) and high light (350 µmol·m^−2^·s^−1^) to examine their enzyme activities. The results showed that the activities of CAT and SOD in *hpr1* mutants were significantly higher than WT under high light intensity, whereas there was no difference between WT and *hpr1* mutants under growth light intensity (Figure 5C), suggesting that the ability of ROS removal was not affected in the mutant. Taken together, these results indicated that high light intensity led to drastic redox reaction harmful to the photosystem; plants lacking HPR1 generated more ROS and had active scavenger enzymes.

### 2.6. The Absence of HPR1 Causes Photorespiratory Metabolism Intermediates Accumulation

**Figure 6:**
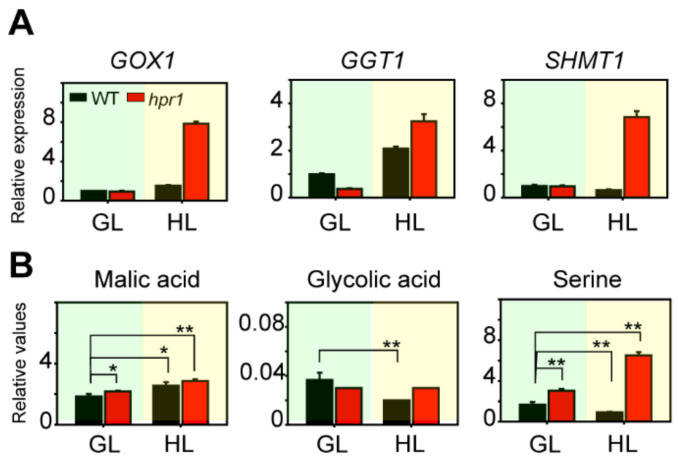
Detection of photorespiratory enzymes and intermediates. (**A**) Expression of photorespiratory enzymes genes in WT and *hpr1* mutants cultivated under growth light (GL, 80 µmol·m^−2^·s^−1^) and high light (HL, 350 µmol·m^−2^·s^−1^). *GOX1*, glycolate oxidase 1; *GGT1*, glutamic acid glyoxalate aminotransferase 1; *SHMT1*, serine hydroxymethyltransferase 1. Data shown are means ± SD, n = 3, with three independent replicates. (**B**) Selected photorespiratory intermediates in WT and *hpr1* mutants. Plants were grown in growth light (GL, 80 µmol·m^−2^·s^−1^) and high light (HL, 350 µmol·m^−2^·s^−1^) with a 16/8 h day/night cycle, and then leaf materials were harvested. Selected metabolites were quantified by GC–MS analysis. Data shown are means ± SD, n = 3, with three independent replicates. Asterisks show significant differences compared to WT under growth light: * *p* < 0.05; ** *p* < 0.01 (Student’s *t*-test).

The major ROS-producing sites during abiotic stress are the chloroplasts, mitochondria, peroxisomes and apoplasts [23,24,25]. Photorespiration is involved in three organelles: the mitochondria, peroxisomes and chloroplasts. Most photorespiratory mutants are chlorotic and show growth retardation [26]. The phenotype of *hpr1* mutants under high light was the same as that under photorespiratory conditions. Therefore, we assumed that high light intensity aggravated photorespiration rates. We performed qRT-PCR to examine the expression levels of some photorespiratory enzymes. *GOX1*, *GGT1* and *SHMT1* regulated photorespiration pathways at the upstream of HPR1. *GOX1* oxidized glycolate into glyoxylate. *GGT1* transformed glyoxylate into glycine. *SHMT1* turned glycine into serine. *GOX1* and *SHMT1* exhibited the same trends both under growth light and high light intensity (Figure 6A). The expression levels of them in *hpr1* mutants were similar to WT when growing under growth light intensity; however, the expression levels of them in *hpr1* mutants were fivefold and 11-fold greater than WT when growing under high light intensity, respectively (Figure 6A). The expression of *GGT1* was somewhat different, because *GGT1* in mutants expressed obviously lower levels than WT under growth light, whereas they were higher than WT under high light intensity (Figure 6A). The intensity of light had no significant influence for wild types, *hpr1* mutants were drastically affected (Figure 6A). Therefore, these results demonstrated that when HPR1 was absent, other photorespiratory enzymes were highly expressed to work against high light stress.

Next, we measured the relative content of metabolic intermediates in photorespiratory bypass [27]. We cultivated WT and *hpr1* mutants under growth light and high light intensity, respectively. Then, the leaves were collected for gas chromatography mass spectrometry (GC–MS) analysis. We observed that it was similar for the contents of malic acid, glycolic acid and serine in WT and *hpr1* mutants under growth light (Figure 6B). Malic acid, glycolic acid and serine were photorespiratory byproducts at the upstream of HPR1. In the photorespiration process, the metabolism of glycolic acid was the key reaction to waste energy. Serine could be an indicator to represent the changes of photorespiration rates. Consistently, the variation tendency of malic acid, glycolic acid and serine in *hpr1* mutants was all higher than WT under high light (Figure 6B). Especially for serine content in *hpr1* mutants, it was 7.9 fold higher than WT under high light intensity (Figure 6B). The existing data indicated that mitochondrial Gly-to-Ser conversion represented potential target sites for the regulation of photorespiration [28]. The high content of serine under high light intensity indicated active photorespiration rates.

## 3. Discussion

As sessile organisms, plants are constantly integrated into complex and everchanging adverse networks of environmental factors that determines their adaptation and survival, such as light, cold, drought, and so on [29]. Sunlight is one of the environmental factors influencing the development and physiology of most living organisms [30]. Specifically, sunlight provides plants with energy for photosynthesis and delivers information about the time of day and season [31]. However, the intensity of light fluctuates, and high light intensity is a kind of stress that is harmful to plants. As an environmental stressor, high light intensity elicits ROS production in plants. The major ROS-producing sites during abiotic stress are the chloroplast, mitochondrion, peroxisome and apoplast [32,33,34,35,36,37]. High light intensity leads to oxidative stress, resulting in photodamage and photoinhibition in chloroplasts [38].

In this study, we focused on three isoenzymes that are associated with photorespiration. We found that HPR1 was located in peroxisomes, while HPR2 and HPR3 were located in the cytoplasm. Then, we investigated the relative levels of tissue expression and promoter *cis*-elements. These experiments demonstrated that all three genes were highly expressed in aboveground tissues such as leaves, stems and reproductive organs, but terrifically expressed at lower levels in underground tissues such as roots. Similarly, promoter analysis revealed that these three genes had many light-responsive *cis*-elements. All of the results above indicated that HPR1, HPR2 and HPR3 probably responded to the light environment and were associated with photosynthesis. Therefore, we next carried out a high light intensity treatment and found that after continuous illumination by high light for 48 hours, the expression of *HPR1*, *HPR2* and *HPR3* reached high levels. We isolated two T-DNA inserted putative mutants, *hpr1* and *hpr3*. The phenotypes of *hpr3* exhibited no difference compared with WT treated with high light; in contrast, *hpr1* looked distinctly etiolated and stunted. To obtain further research on how HPR1 reacted to the high light environment, the vector was transferred into *hpr1* mutants by the floral-dip method, and we successfully obtained complements for two lines with different gene expression. The complementary lines could rescue some phenotypes under high light illumination. The following was to detect the physiological data of *hpr1* mutants. When growing in a growth light environment, the fresh weight, chlorophyll contents and photosynthetic efficiency of *hpr1* mutants were the same with those of WT. When growing in a chamber with high light intensity, the fresh weight, chlorophyll contents and photosynthetic efficiency of *hpr1* mutants were significantly lower than those of WT. For complementary lines, the basic physiological data could also be rescued. We confirmed that HPR1 responded to high light intensity and the lack of HPR1 caused serious growth defects under high light.

Because of decreased photosynthetic efficiency and etiolated characteristics, we further explored the composition and structure of chloroplasts. As a classical method of photosynthesis measurement, chlorophyll fluorescence can reflect almost all changes in the photosynthesis process. Y(II) represents the actual and real-time light-energy conversion efficiency and quantum yield of PSII. Electron transport rates (ETR) represent the relative linear electron flow rate through PSII. Undoubtedly, PSII of the *hpr1* mutants suffered greatly from excess light intensity. Nevertheless, the assembly of thylakoid photosynthetic machinery of *hpr1* seedlings was not significantly different from that of WT. And the abundance of PSⅠ and PSII proteins in the *hpr1* mutants were also similar to WT.

Light intensity exceeding the energetic demand of photosynthesis leads to damage to the photosynthetic machinery, especially to PSII, and thus caused photoinhibition [31]. Plants have evolved rapidly deployed responses under high light environments to prevent photosynthetic oxidative stress in chloroplasts [39], which are coupled with a PSII repair system [40,41]. We performed photodamage and photorepair experiments; apparently, the absence of HPR1 impaired the rates of photosystem repair.

When these avoidance mechanisms are not sufficient, plants have to deal with the excess light absorbed by the photosynthetic pigments that produce ROS and induce oxidative stress. After treating with a light intensity of 1000 µmol·m^−2^·s^−1^, we discovered more ROS in propoplasts infected with H_2_DCFDA by confocal microscopy. To avoid oxidative stress, plants have evolved several ROS scavenging enzymatic and nonenzymatic antioxidant defense systems [42]. Representative antioxidant enzymes include catalase (CAT) and superoxide dismutase (SOD). The results implied highly active ROS removal under high light. HPR1 has been reported to be a photorespiratory enzyme, and one of the crucial functions of photorespiration is ROS generation. Some photorespiratory enzymes were highly expressed such as *GOX1*, *GGT1* and *SHMT1*, and some intermediates accumulated a lot such as malic acid, glycolic acid and serine in *hpr1* mutants. Taking all of the results into account, we hypothesized that, faced with an extreme environment like high light intensity, the lacking of HPR1 aggravated photorespiratory rates and ROS generation, plants accumulated too much ROS and harmful metabolic intermediates, so that the *hpr1* mutants displayed serious growth retardation and etiolation as well as damage to PSII in chloroplasts.

It has already been reported that HPR1 is induced by light [43]. In our research, we primarily found that HPR1 was physiologically responsive to high light intensity, and the lack of HPR1 significantly affected the photosynthetic efficiency and slowed the recovery rates of the photosystem, and caused much ROS and serious photorespiratory function when exposed to high light intensity. Additionally, we first discovered the location of HPR1 in *Arabidopsis* under confocal microscopy. The uncovered association among ROS, photorespiration and high light stress indicated that HPR1 has important roles in developing photosynthetic energy utilization and crop productivity. Furthermore, we could improve our understanding of plants against extreme environments. Nevertheless, the mechanisms of how HPR1 aggravates photorespiration rates and ROS generation remain unclear and are also a challenge to solve.

## 4. Materials and Methods

### 4.1. Plant Materials, Growth Conditions and Chemicals

Arabidopsis thaliana ecotype Columbia-0 was used in this study as a wild type (WT) control. The T-DNA insertion mutant lines (SALK_143584, locus At1g68010 and SALK_019014, locus At1g12550) were obtained from the Arabidopsis Resource Center (Columbus, OH, USA). For soil-grown plants, sown seeds were cold treated at 4 °C for 2 days and then transferred to the indicated growth conditions (22 °C, 16-h light/8-h dark, 80 µmol·m^−2^·s^−1^).

We performed experiments with three biological replicates. The significance analysis between WT and *hpr1* mutants were performed by a student’s *t*-test.

### 4.2. Chlorophyll Fluorescence and Chlorophyll Content Measurements

Chlorophyll fluorescence parameters were measured by Fluor Cam800-C (Photon System Instruments, Prague, Czech Republic) after 30-min dark adaptation. For pigment extraction, plant tissues (approximately 0.2 g of fresh weight) were immersed in 80% acetone overnight. Spectrophotometric quantification was carried out in NanoDrop2000 (Thermo Fisher, Waltham, MA, USA) using the following calculations:

Chla=12.21∗A663−2.81∗A645 and Chlb=20.13∗A645−5.03∗A663 (µg/mL).

### 4.3. RNA Extraction, Reverse Transcription and Quantitative RT–PCR Assays

Following the protocol of the manufacturer of TRIzol reagent (Invitrogen, Carlsbad, CA, USA), total RNA was extracted. RNA was reversely transcribed into cDNA with the Prime Script™ RT Reagent Kit (TaKaRa, Tokyo, Japan). For the quantitative real-time polymerase chain reaction (quantitative RT–PCR) assay, we used 7300 plus Real-Time PCR system (Applied Biosystems, Foster City, CA, USA) with SYBR Premix Ex Taq (TaKaRa, Tokyo, Japan). The Actin gene was used as the endogenous control. Relative expression was calculated by using the formula 2^−ΔΔCt^.

### 4.4. Blue-Native PAGE and Second-Dimension SDS–PAGE

Blue-native PAGE was performed as described [44]. Chloroplast thylakoid proteins were quantified to 15 μg of chlorophyll and then loaded on native gradient page gels (4% to 16% acrylamide) for electrophoresis for 6–8 h. For the second-dimensional SDS–PAGE, lanes of BN-PAGE gel were sliced cautiously and then submerged in 2X SDS loading buffer containing 2% β-mercaptoethanol for 15 min at 75 °C. The gel was embedded on top of 12% SDS–PAGE for electrophoresis, and then the proteins were stained by Coomassie Brilliant Blue R250.

### 4.5. Western Blot

The protein samples of thylakoid membranes were denatured for 15 min at 95 °C, separated by 12% SDS–PAGE, and then transferred to nitrocellulose membranes. After blocking with 5% nonfat dried milk, the membranes were reacted with antibodies including PSⅠ and PSII proteins in this study, finally were detected using an electrochemiluminescence kit (Beyotime, Shanghai, China), then analyzed by chemiluminescence imaging (Bio–Rad, Hercules, CA, USA).

### 4.6. Complementation of the hpr1

The *HPR1* coding region was amplified from *Arabidopsis* cDNA. The aimed *HPR1* fragment was cloned into transient expression vector pEarley101 under control of the cauliflower mosaic virus 35S promoter. The plasmids were transferred into *Agrobacterium tumefaciens* (strain GV3101) by elec-transformation. The cells were transformed into *hpr1* mutant plants using the floral dip method. The transformants were selected on ^1^/_2_ MS medium containing 8 mg/L Basta.

### 4.7. Detection of ROS

We detected the fluorescence of DCF to determine reactive oxygen species production. *Arabidopsis* protoplasts of WT and *hpr1* were incubated with H_2_DCFDA (Sigma) at a final concentration of 5 µM, and then these protoplasts were treated for 0 and 30 min under high light of 1000 µmol·m^−2^·s^−1^. H_2_DCFDA was oxidized into DCF. Intracellular ROS production and distribution, as well as chloroplast autofluorescence, were visualized under a confocal laser scanning microscope. The relative DCF was measured with fluorescence intensity at 525 nm, and chloroplast autofluorescence was visualized at 650 nm. All these were excited at a wavelength of 488 nm.

### 4.8. Measurement of Antioxidant Enzyme Activity

We carried out this experiment as described [45]. We collected *Arabidopsis thaliana* leaves for four weeks. The fresh samples were pulverized in liquid nitrogen and extracted with ice-cold phosphate buffer (0.05 M, pH 7.8). The homogenate was then centrifuged for 20 min (11,000× *g*, 4 °C). The activities of catalyze (CAT) and superoxide dismutase (SOD) enzymes were determined in the supernatant. 0.2 mol/L H_2_O_2_ solution was added, and the absorbance value at 240 nm was measured on the UV spectrophotometer immediately. The absorbance value was read every 30 s for 3 min. The decline in absorbance per minute at 240 nm corresponds to a unit of CAT activity.

The total reaction liquid included 0.05 M phosphate buffer, 130 mM Met solution, 750 µM NBT solution, 100 µM EDTA-Na_2_, 20 µM riboflavin and supernatant. The reaction solution was mixed and treated under light illumination for 20 min. One SOD enzyme activity unit is defined as 50% inhibition of NBT reduction at 560 nm [46].

### 4.9. Isolation of Arabidopsis Mesophyll Protoplasts

We isolated *Arabidopsis thaliana* protoplasts from four-week-old *Arabidopsis* wild types [47]. The leaves were cut into 0.5–1 mm strip and digested in 10 mL enzyme solution. The purified protoplasts were suspended in W5 solution (2 mM MES pH 5.7, 154 mM NaCl, 125 mM CaCl_2_ and 5 mM KCl). Centrifugation was performed at 100× *g* for 5 min. The supernatant was slowly sucked up, and the precipitate was re-suspended with 10 mL W5 solution, and then left on ice for 30 min. Centrifugation was performed at 100× *g* for 5 min. The protoplast was obtained by adding MMG Solution (0.2 M MES, 0.8 M Mannitol, 1 M MgCl_2_).

### 4.10. Subcellular Localization

cDNAs of *HPR1*, *HPR2* and *HPR3* were subcloned into pUGW41 and pUGW42 using Gateway technology. The WT protoplasts prepared from mesophyll cells were transfected with the plasmids. After 16–20 h of transfection, protoplasts were observed using a confocal laser scanning microscope (TCS SP8). Excitation wavelengths were 473 nm for GFP and 635 nm for chlorophyll. Emissions were collected from 490 to 540 nm for GFP and 660 to 710 nm for chlorophyll.

### 4.11. Analysis of PSII Photoinhibition and Recovery

To determine the *Arabidopsis thaliana* sensitivity of PSII to high light stress, we measured *F_v_*/*F_m_* as an indicator and used leaves of four-week-old mutant and WT plants with or without lincomycin. For photodamage, the detached rosette leaves were exposed to 1000 µmol·m^−2^·s^−1^ light for 6 h. Photorecovery was followed under normal light conditions (60 µmol·m^−2^·s^−1^) for 6 h.

### 4.12. Promoter Analysis

Sequences of 2-kb fragments upstream of *Arabidopsis HPR1*, *HPR2* and *HPR3* genes were downloaded from Tair (Tair address: https://www.arabidopsis.org/ accessed on 17 December 2021). The regulatory *cis*-acting elements of *Arabidopsis HPR1*, *HPR2* and *HPR3* genes were then analyzed using PlantCARE (PlantCARE address: http://bioinformatics.psb.ugent.be/webtools/plantcare/html/ accessed on 17 December 2021).

### 4.13. Chlorophyll Fluorescence of PSII with PAM-2500 (Walz, Effeltrich, Germany)

Chlorophyll fluorescence inductive curve and light response curve measurements were carried out on PAM-2500 fluorimeter. The samples under growth light and high light were kept dark for 30 min, then we turned on a measuring light, and F0 was recorded. A saturation Pulse was given to mark down Fm. Photochemical and nonphotochemical parameters were calculated as described [48].

$$Y\left(\mathrm{II}\right)=\Delta F/{\mathrm{Fm}}^{\prime }=\left({\mathrm{Fm}}^{\prime }-F\right)/{\mathrm{Fm}}^{\prime }.\;\mathrm{ETR}\left(\mathrm{II}\right)=\;\mathrm{PAR}*Y\left(\mathrm{II}\right)*0.84*0.5.$$

The measurement of light response curves could be performed directly under light.

### 4.14. Metabolite Extraction, Gas Chromatography–Time of Flight–Mass Spectrometry Analysis

Metabolite extraction and analysis were performed essentially as described [49]. Three-week-old wild types and *hpr1* mutants were treated by 80 µmol·m^−2^·s^−1^ and 350 µmol·m^−2^·s^−1^ for one week, respectively. Then we collected leaves of these sample plants and rapidly froze them in liquid nitrogen. We then added 1.4 mL of 100% methanol and 0.5 mL Ribitol (0.2 mg/mL) (yuanye biology, Shanghai, China) into the mashed leaves, and centrifuged them at 12,000 rpm, 4 °C condition for 15 min. The supernatant was concentrated and then derivatized with MSTFA reagent (Sigma-Aldrich, Saint Louis, MO, USA). Finally, the extractive was transferred into glass vials suitable with Thermo Scientific GC-MS (ISQ GC/MS), and analyzed by Xcalibur.

## Data Availability

Data are available on request to the corresponding author.

## Supplementary Materials

The following supporting information can be downloaded at: https://www.mdpi.com/article/10.3390/ijms23084444/s1.

## References

[1] Mittler R. (2002). Oxidative stress, antioxidants and stress tolerance. Trends Plant Sci..

[2] Apel K., Hirt H. (2004). Reactive oxygen species: Metabolism, oxidative stress, and signal transduction. Annu. Rev. Plant Biol..

[3] Mahajan S., Tuteja N. (2005). Cold, salinity and drought stresses: An overview. Arch. Biochem. Biophys..

[4] Gill S.S., Khan N.A., Anjum N.A., Tuteja N. (2011). Amelioration of Cadmium Stress in Crop Plants by Nutrient Management: Morphological, Physiological and Biochemical Aspects. Plant Stress.

[5] Munns R., Tester M. (2008). Mechanisms of salinity tolerance. Annu. Rev. Plant Biol..

[6] Zorov D.B., Juhaszova M., Sollott S.J. (2006). Mitochondrial ROS-induced ROS release: An update and review. Biochim. Biophys. Acta Bioenerg..

[7] Gill S.S., Tuteja N. (2010). Reactive oxygen species and antioxidant machinery in abiotic stress tolerance in crop plants. Plant Physiol. Biochem..

[8] Triantaphylidès C., Havaux M. (2009). Singlet oxygen in plants: Production, detoxification and signaling. Trends Plant Sci..

[9] Foyer C.H., Bloom A.J., Queval G., Noctor G. (2009). Photorespiratory Metabolism: Genes, Mutants, Energetics, and Redox Signaling. Annu. Rev. Plant Biol..

[10] Pieuchot L., Jedd G. (2012). Peroxisome Assembly and Functional Diversity in Eukaryotic Microorganisms. Annu. Rev. Microbiol..

[11] Hu J., Baker A., Bartel B., Linka N., Mullen R.T., Reumann S., Zolman B.K. (2012). Plant Peroxisomes: Biogenesis and Function. Plant Cell.

[12] Bauwe H., Hagemann M., Fernie A.R. (2010). Photorespiration: Players, partners and origin. Trends Plant Sci..

[13] Timm S., Florian A., Jahnke K., Nunes-Nesi A., Fernie A.R., Bauwe H. (2011). The Hydroxypyruvate-Reducing System in Arabidopsis: Multiple Enzymes for the Same End. Plant Physiol..

[14] Timm S., Nunes-Nesi A., Pärnik T., Morgenthal K., Wienkoop S., Keerberg O., Weckwerth W., Kleczkowski L.A., Fernie A.R., Bauwe H. (2008). A Cytosolic Pathway for the Conversion of Hydroxypyruvate to Glycerate during Photorespiration in Ara-bidopsis. Plant Cell.

[15] Mano S., Hayashi M., Kondo M., Nishimura M. (1997). Hydroxypyruvate reductase with a carboxy-terminal targeting signal to microbodies is expressed in Arabidopsis. Plant Cell Physiol..

[16] Zushi K., Matsuzoe N. (2017). Using of chlorophyll a fluorescence OJIP transients for sensing salt stress in the leaves and fruits of tomato. Sci. Hortic..

[17] Guidi L., Lo Piccolo E., Landi M. (2019). Chlorophyll Fluorescence, Photoinhibition and Abiotic Stress: Does It Make Any Difference the Fact to Be a C3 or C4 Species?. Front Plant Sci..

[18] Demmig-Adams B., Cohu C.M., Muller O., Adams W.W. (2012). Modulation of photosynthetic energy conversion efficiency in nature: From seconds to seasons. Photosynth. Res..

[19] Osmond C.B. (1981). Photorespiration and photoinhibition: Some implications for the energetics of photosynthesis. Biochim. Biophys. Acta (BBA)-Rev. Bioenerg..

[20] Osmond C.B., Grace S.C. (1995). Perspectives on photoinhibition and photorespiration in the field: Quintessential inefficiencies of the light and dark reactions of photosynthesis?. J. Exp. Bot..

[21] Wingler A., Lea P.J., Quick W.P., Leegood R.C. (2000). Photorespiration: Metabolic pathways and their role in stress protection. Philos. Trans. R. Soc. B Biol. Sci..

[22] Takahashi S., Bauwe H., Badger M. (2007). Impairment of the Photorespiratory Pathway Accelerates Photoinhibition of Photo-System II by Suppression of Repair but Not Acceleration of Damage Processes in *Arabidopsis*. Plant Physiol..

[23] Dietzel L., Bräutigam K., Pfannschmidt T. (2008). Photosynthetic acclimation: State transitions and adjustment of photosystem stoichiometry—Functional relationships between short-term and long-term light quality acclimation in plants. FEBS J..

[24] Vainonen J.P., Sakuragi Y., Stael S., Tikkanen M., Allahverdiyeva Y., Paakkarinen V., Aro E., Suorsa M., Scheller H.V., Vener A.V. (2008). Light regulation of CaS, a novel phosphoprotein in the thylakoid membrane of *Arabidopsis thaliana*. FEBS J..

[25] Pesaresi P., Hertle A., Pribil M., Kleine T., Wagner R., Strissel H., Ihnatowicz A., Bonardi V., Scharfenberg M., Schneider A. (2009). *Arabidopsis* STN7 Kinase Provides a Link between Short- and Long-Term Photosynthetic Acclimation. Plant Cell.

[26] Eisenhut M., Roell M., Weber A.P.M. (2019). Mechanistic understanding of photorespiration paves the way to a new green revolution. New Phytol..

[27] Peterhansel C., Horst I., Niessen M., Blume C., Kebeish R., Kürkcüoglu S., Kreuzaler F. (2010). Photorespiration. Arab. Book.

[28] Reinholdt O., Schwab S., Zhang Y., Reichheld J.-P., Fernie A.R., Hagemann M., Timm S. (2019). Redox-Regulation of Photorespiration through Mitochondrial Thioredoxin o1. Plant Physiol..

[29] Lee H.Y., Back K. (2018). Melatonin induction and its role in high light stress tolerance in *Arabidopsis thaliana*. J. Pineal Res..

[30] Galvão V.C., Fankhauser C. (2015). Sensing the light environment in plants: Photoreceptors and early signaling steps. Curr. Opin. Neurobiol..

[31] Roeber V.M., Bajaj I., Rohde M., Schmülling T., Cortleven A. (2021). Light acts as a stressor and influences abiotic and biotic stress responses in plants. Plant Cell Environ..

[32] Dietz K.-J., Turkan I., Krieger-Liszkay A. (2016). Redox- and Reactive Oxygen Species-Dependent Signaling into and out of the Photosynthesizing Chloroplast. Plant Physiol..

[33] Gilroy S., Białasek M., Suzuki N., Górecka M., Devireddy A.R., Karpiński S., Mittler R. (2016). ROS, Calcium, and Electric Signals: Key Mediators of Rapid Systemic Signaling in Plants. Plant Physiol..

[34] Huang S., Van Aken O., Schwarzländer M., Belt K., Millar A.H. (2016). The Roles of Mitochondrial Reactive Oxygen Species in Cellular Signaling and Stress Response in Plants. Plant Physiol..

[35] Kerchev P., Waszczak C., Lewandowska A., Willems P., Shapiguzov A., Li Z., Alseekh S., Mühlenbock P., Hoeberichts F.A., Huang J. (2016). Lack of GLYCOLATE OXIDASE1, but Not GLYCOLATE OXIDASE2, Attenuates the Photorespiratory Phenotype of CATALASE2-Deficient Arabidopsis. Plant Physiol..

[36] Rodríguez-Serrano M., Romero-Puertas M.C., Sanz-Fernández M., Hu J., Sandalio L.M. (2016). Peroxisomes Extend Peroxules in a Fast Response to Stress via a Reactive Oxygen Species-Mediated Induction of the Peroxin PEX11a. Plant Physiol..

[37] Takagi D., Takumi S., Hashiguchi M., Sejima T., Miyake C. (2016). Superoxide and Singlet Oxygen Produced within the Thylakoid Membranes Both Cause Photosystem I Photoinhibition. Plant Physiol..

[38] Ganguly D.R., Crisp P.A., Eichten S.R., Pogson B.J. (2018). Maintenance of pre-existing DNA methylation states through recurring excess-light stress. Plant Cell Environ..

[39] Hodges M., Barber J. (1983). Photosynthetic adaptation of pea plants grown at different light intensities: State 1-State 2 transitions and associated chlorophyll fluorescence changes. Planta.

[40] Adam Z., Clarke A.K. (2002). Cutting edge of chloroplast proteolysis. Trends Plant Sci..

[41] Sun X., Peng L., Guo J., Chi W., Ma J., Lu C., Zhang L. (2007). Formation of DEG5 and DEG8 complexes and their involvement in the degradation of photodamaged photosystem II reaction center D1 protein in *Arabidopsis*. Plant Cell..

[42] Mittler R., Vanderauwera S., Gollery M., Van Breusegem F. (2004). Reactive oxygen gene network of plants. Trends Plant Sci..

[43] Wang Y., Beaith M., Chalifoux M., Ying J., Uchacz T., Sarvas C., Griffiths R., Kuzma M., Wan J., Huang Y. (2009). Shoot-Specific Down-Regulation of Protein Farnesyltransferase (α-Subunit) for Yield Protection against Drought in Canola. Mol. Plant.

[44] Kügler M., Jansch L., Kruft V., Schmitz U.K., Braun H.-P. (1997). Analysis of the chloroplast protein complexes by blue-native polyacrylamide gel electrophoresis (BN-PAGE). Photosynth. Res..

[45] Chen G., Li S. (2016). Plant Physiology Experiment.

[46] Feng R., Wei C. (2012). Antioxidative mechanisms on selenium accumulation in *Pteris vittata* L., a potential selenium phytoremediation plant. Plant Soil Environ..

[47] He P., Shan L., Sheen J. (2007). The use of protoplasts to study innate immune responses. Methods Mol Biol..

[48] Maxwell K., Johnson G.N. (2000). Chlorophyll fluorescence—A practical guide. J. Exp. Bot..

[49] Lisec J., Schauer N., Kopka J., Willmitzer L., Fernie A.R. (2006). Gas chromatography mass spectrometry-based metabolite profiling in plants. Nat. Protoc..

