# Supplementary material for: HPR1 Is Required for High Light Intensity Induced Photorespiration in Arabidopsis thaliana
Source: Int J Mol Sci. 2022 Apr 18;23(8):4444. doi: 10.3390/ijms23084444 (PMC9030206; doi:10.3390/ijms23084444)
Supplement: Supplementary file 1 [file ijms-23-04444-s001.zip › ijms-1667936-supplementary.pdf]

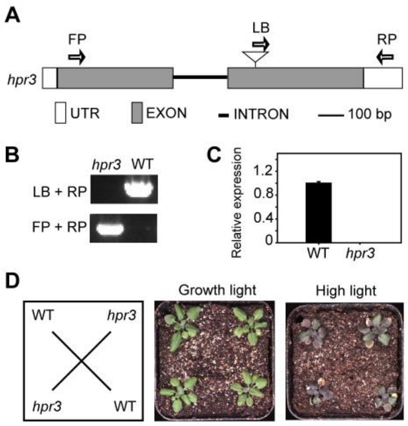

Supplemental Figure S1. Identification of the *hpr3* mutant. (**A**) Genomic structure model of *HPR3*. Gray rectangles show the open reading frame, black line shows intron, white rectangles show untranslated regions, and triangles represent T-DNA insertion. (**B**) PCR analysis of genomic DNA from the WT and *hpr3-1* plants. LB, PR and FP indicate primers which locations are shown in ***A***. (**C**) The expression of *HPR3* in WT and *hpr3* plants was analyzed by qRT-PCR. (**D**) Phenotypes of WT and *hpr3* with different light intensity treatments. WT and *hpr3* grown under growth light of 80 μmol·m^{-2}·s^{-1} for 3 weeks, were treated under growth light with 80 μmol·m^{-2}·s^{-1} and high light with 350 μmol·m^{-2}·s^{-1} for another week, respectively.
